# Efficacy and safety of mechanical thrombectomy in the posterior cerebral circulation—a single center study

**DOI:** 10.1038/s41598-024-57963-6

**Published:** 2024-04-02

**Authors:** Michał Borończyk, Mikołaj Kuźniak, Agnieszka Borończyk, Amadeusz Żak, Łukasz Binek, Anna Wagner-Kusz, Anetta Lasek-Bal

**Affiliations:** 1 Students’ Scientific Association, Department of Neurology, Faculty of Health Sciences in Katowice, Medical University of Silesia, 40-055 Katowice, Poland; 2 Department of Neurology, Faculty of Health Sciences in Katowice, Medical University of Silesia, Katowice, Poland; 3 Upper-Silesian Medical Centre, Silesian Medical University in Katowice, Katowice, Poland

**Keywords:** Stroke, Mechanical thrombectomy, Outcomes, Posterior circulation, Anterior circulation, Cerebrovascular disorders, Stroke

## Abstract

Mechanical thrombectomy (MT) is the current standard treatment for strokes in the anterior cerebral circulation (AMT) and has recently been proven to be beneficial in the posterior circulation strokes (PMT). Our study aims to evaluate parameters for favorable outcomes in PMT-patients and to compare the clinical characteristics of individuals who received AMT and PMT. For this purpose, we confronted AMT and PMT-receipients and performed a multivariate regression analysis to assess the influence of factors on favorable outcomes in the study group and in the AMT and PMT subgroups. When analysing 623 MT-patients, those who received PMT had significantly lower admission National Institutes of Health Stroke Scale (NIHSS) scores (9 vs. 13; p < 0.001) and 24 h post-MT (7 vs. 12; p = 0.006). Key parameters influencing the favorable outcomes of PMT at discharge and at 90th day include: NIHSS scores (OR: 0.865, 95% CI: 0.813–0.893, and OR: 0.900, 95% CI: 0.861–0.925), MT time (OR: 0.993, 95% CI: 0.987–0.998 and OR: 0.993, 95% CI: 0.990–0.997), and leukocytosis (OR: 0.961, 95% CI: 0.928–0.988 and OR: 0.974, 95% CI: 0.957–0.998). Different clinical profiles exist between AMT and PMT-recipients, with the neurological status post-MT being decisive for the prognosis. Several factors play an important role in predicting outcome, especially in the PMT group.

## Introduction

Mechanical thrombectomy (MT) is a well-studied method and, in combination with intravenous thrombolysis (IVT), represents the current gold standard in the treatment of ischemic stroke in the anterior cerebral vessels within a 6-h window^1^. Although there is limited data available on the efficacy and safety of MT in the posterior vasculature, recent studies have demonstrated the effectiveness of this procedure compared to the best non-invasive treatment^2,3^. Acute ischemic strokes in the posterior cerebral vessels are estimated to account for 13.5–20% of all strokes^4–6^.

When comparing the efficacy, safety, and favorable outcomes of MT in the anterior (AMT) and posterior (PMT) circulation, studies often yield different results. While PMT is associated with higher mortality and a lower rate of functional independence on the 90th day^6^, successful reperfusion and favorable outcomes may be comparable in both the AMT and PMT groups^7–9^. Jahan et al. demonstrated that PMT had similar rates of functional independence after 90 days, successful reperfusion, and mortality compared to AMT^10^. The authors point out that by selecting patients appropriately for MT, comparable treatment results can be achieved^10,11^. Approximately 1% of ischemic strokes are associated with basilar artery occlusion, which is the most severe form of stroke and is related with a high mortality rate^12^. Likewise, in the studied patient group, a favorable functional outcome can be achieved significantly more often with IVT and successful reperfusion treatment combined^13^.

The primary objective of this study was to evaluate the efficacy and safety of PMT, as well as to identify factors affecting patients' functional status at discharge and 90 days after PMT. Additionally, we wanted to analyze the factors influencing PMT outcomes in relation to those affecting MT outcomes in the entire patient group, encompassing both PMT and AMT recipients.

## Materials and methods

### Patient population

We performed the retrospective study, including an analysis of selected data from patients who suffered from ischemic stroke between March 1, 2019, and January 31, 2022, and were treated by MT (stent‐retriever or contact aspiration as a first‐line technique). Participants in the study were recruited among patients treated at the Department of Neurology with Stroke Subdivision of the Upper Silesian Medical Centre, Medical University of Silesia in Katowice, Poland. Data were obtained from the hospital database.

This study was reviewed and approved by the local ethics committee, the “Bioethics Committee of the Medical University of Silesia in Katowice”, in accordance with local and regional laws. Therefore, it was performed in accordance with the ethical standards of the 1964 Declaration of Helsinki and its subsequent amendments. Due to the retrospective nature of the study, the need to obtain informed consent was waived by the aforementioned local bioethics committee.

### Clinical characteristics of the cohort

We collected the following patient data: baseline demographic data (age, sex), clinical data (stroke severity as measured by the National Institutes of Health Stroke Scale (NIHSS) on admission; before MT-NIHSS1, and on the second day of stroke-NIHSS2), risk factor profile [atrial fibrillation (AF), hypertension, diabetes mellitus (DM), nicotinism, dyslipidemia, atherosclerosis], radiological data (location of artery occlusion), time from onset of symptoms to MT, duration and efficacy of MT (measured by the modified thrombolysis in cerebral infarction (mTICI) scale, with values of 2b–3 defined as successful and 0–2a as lack of effective reperfusion), whether IVT was administrated (alteplase in dose of 0.9 mg/kg intravenously), laboratory tests [C-reactive protein (CRP), leukocytosis] and whether a decompressive hemicraniectomy (DH) was performed. The main criteria of DH were as follows: Glasgow coma score > 5, NIHSS score ≥ 16, computed tomography scan of the head showing ischemic changes corresponding to more than 2/3 of the MCA territory, and intervention possible within 48 h after MT. Intracranial hemorrhage (ICH) events, including hemorrhagic conversions of ischemic stroke, were classified according to the European Cooperative Acute Stroke Study scale (ECASS II). In the statistical analyses only parenchymal hematoma type 2 (PH2) was included (> 30% of infarct zone, substantial mass effect attributable to the hematoma). Patient functional outcomes were measured using the modified Rankin Scale (mRS) and were obtained at the following time points: at discharge from the department and on the 90th day after the procedure. A favorable outcome was defined as a maximum of 2 points on the mRS. All factors mentioned above were compared between the AMT and PMT groups.

We divided the patients into two main groups: those treated with MT in the anterior cerebral arteries (AMT group), including the internal carotid artery (ICA), the middle cerebral artery (MCA) or the anterior cerebral artery (ACA), and those treated with MT in the posterior circulation (PMT group), including the vertebral artery (VA), the basilar artery (BA) or the posterior cerebral artery (PCA).

### Statistical analysis

Continuous variables were expressed as mean ± standard deviation (SD) or median and range. Numbers and percentages were used to represent categorical variables. The normality of distribution was assessed using the Shapiro–Wilk test. The Student t-test and the Mann–Whitney U-test were used to determine the significance of differences between the groups for continuous variables, while the Chi-square test or Fisher's accuracy test was used in univariate analyzes for categorical variables. Statistical significance was defined as p-value > 0.05. Ordinal regression analysis was used for full granular mRS score (mRS 0–2 vs. 3–6) and the binary logistic model was applied. In the next step, significant factors were further analyzed using binary logistic regression (analysis with the backward stepwise method).

Subsequently, the impact of clinical factors on a favorable prognosis was analyzed in both the entire study population and subgroups (patients after AMT and after PMT) using ordinal univariate logistic regression analysis. Statistical significance was set at p-value > 0.05. In the next step, significant factors were further analyzed using ordinal multivariate logistic regression analysis with the backward stepwise method. This approach aimed to construct the most effective model for predicting favorable outcomes, while excluding factors affected by multicollinearity, excessively correlated variables, and interactions between variables. Again, statistical significance was defined at p-value > 0.05. Associations are presented as odds ratios (OR) with a 95% confidence interval (CI). The analysis was conducted using the Statistica 13.3 program.

## Results

During the study period, 2036 patients were hospitalized due to ischemic stroke at the Neurology Department in Upper-Silesian Medical Centre of the Medical University of Silesia in Katowice. The study included 623 stroke patients who underwent MT, with 57 (9.15%) in the PMT group and 566 (90.85%) in the AMT group. The stent‐retriever treatment was used in 379 (60.84%) patients as a first‐line MT technique and contact aspiration was used in 244 (39.16%) patients.

The average age of the patients was 66.9 [20–92] years, with 44.94% of them being female. Using a comparative analysis, we evaluated the clinical and radiological parameters between patients with AMT vs. PMT. The groups differed significantly in terms of age (median in AMT group = 70 vs. in PMT group = 63; p = 0.001), gender (46.82% women in AMT group vs. 26.32% in PMT group; p = 0.002), NIHSS1 (median in AMT group = 13 vs. in PMT group = 9 ; p < 0.001), and NIHSS2 (median in AMT group = 12 vs. in PMT group = 7; p = 0.007). There were no significant differences in mortality rates at any of time points. The complete group base characteristics are shown in Table 1.

**Table 1:** Baseline characteristics of patients with MT in AMT and PMT.

	All patients	AMT	PMT	p-value
Age (mean, median, range)	66.99, 69 [20–92]	67.56, 70 [20–92]	61, 63 [23–84]	0.001^1^
Women (n, %)	280 (44.94%)	265 (46.82%)	15 (26.32%)	0.002^2^
Hospitalization time (mean, median, range)	12.23, 9 [0–70]	12.46, 9 [1–70]	10.57, 8 [0–55]	0.32^1^
NIHSS1 (mean, median, range)	12.87, 12 [0–30]	13.15, 13 [1–30]	10.54, 9 [0–43]	< 0.001^1^
NIHSS2 (mean, median, range)	12.16, 12 [0–30]	12.33, 12 [0–30]	10.71, 7 [0–43]	0.007^1^
mRS at discharge (0–2) (n, %)	156 (25.04%)	139 (24.56%)	17 (29.82%)	0.38^2^
mRS after 1 month (0–2) (n, %)	252 (40.45%)	226 (39.93%)	26 (45.61%)	0.40^2^
mRS after 3 months (0–2) (n, %)	322 (52.1%)	292 (51.96%)	30 (53.57%)	0.82^2^
AF (n, %)	237 (38.04%)	221 (39.05%)	16 (28.07%)	0.10^2^
Arterial hypertension (n, %)	439 (70.3%)	402 (71.02%)	37 (64.91%)	0.33^2^
DM (n, %)	158 (25.36%)	143 (25.27%)	16 (28.07%)	0.64^2^
Atherosclerosis (n, %)	234 (37.56%)	216 (38.16%)	18 (31.58%)	0.32^2^
Smoking (n, %)	165 (26.48%)	146 (38.5%)	19 (44.18%)	0.47^2^
Dyslipidemia (n, %)	233 (37.52%)	208 (36.75%)	26 (45.61%)	0.18^2^
Thrombolysis (n, %)	392 (62.92%)	353 (62.37%)	40 (70.18%)	0.24^2^
mTICI 2b-3 (n, %)	449 (72.07%)	414 (73.14%)	39 (68.42%)	0.44^2^
Time from symptoms to MT (mean, median, range)	268.7, 270 [5- 870]	267.16, 270 [5–870]	286.1, 270 [98–780]	0.32^1^
MT time (mean, median, range)	98.22, 90 [30–270]	98.16, 90 [30–270]	102, 100 [45–190]	0.29^1^
Mortality at discharge (n, %)	111 (17.82%)	98 (17.31%)	13 (22.81%)	0.30^2^
Mortality after 1 month (n, %)	130 (20.87%)	116 (20.49%)	14 (24.56%)	0.47^2^
Mortality after 3 months (n, %)	145 (23.27%)	129 (22.79%)	16 (28.07%)	0.37^2^
CRP concentration (mean, median, range)	20.59, 8.7 [3–254]	20.88, 8.9 [3–254]	17.71, 8 [4–127]	0.81^1^
WBC count (> 10,000) (n, %)	341 (54.74%)	308 (54.42%)	33 (57.89%)	0.61^2^
DH (n, %)	27 (4.33%)	24 (4.24%)	3 (5.26%)	0.98^3^
ICH (n, %)	109 (17.5%)	101 (17.84%)	8 (14.04%)	0.47^2^

*AMT* anterior mechanical thrombectomy, *PMT* posterior mechanical thrombectomy, *NIHSS1* National Institutes of Health Stroke Scale at admission, *NIHSS2* National Institutes of Health Stroke Scale 24 h after intervention, *mRS* modified Rankin Scale, *AF* atrial fibrillation, *DM *diabetes mellitus; *mTICI 2b–3* modified Thrombolysis In Cerebral Infarction scale 2b–3, *CRP *C-reactive protein, *WBC* white blood cells, *DH* decompressive hemicraniectomy, *ICH* intracranial hemorrhage.

^1^U Mann- Whitney test, ^2^Pearson’s Chi^2^ test, ^3^Yates’ Chi^2^ test.

**Figure 1:**
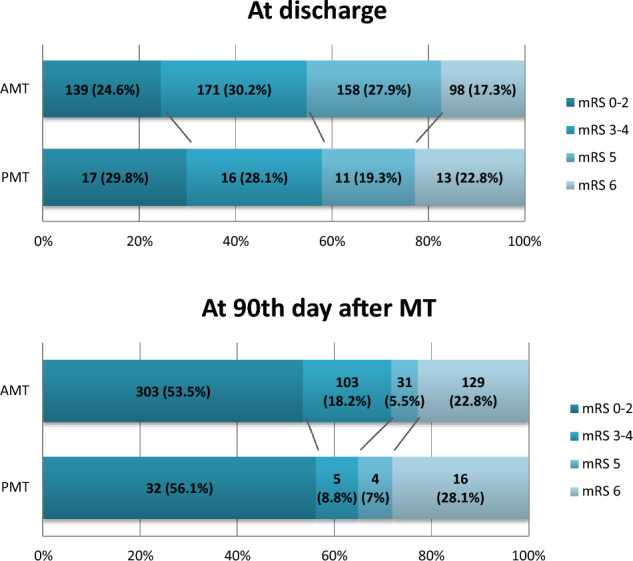
Comparison of outcomes in patients with PMT and AMT at discharge and at 90th day of stroke. *AMT* anterior mechanical thrombectomy, *PMT* posterior mechanical thrombectomy, *MT* mechanical thrombectomy, *mRS* modified Rankin Scale.

Among all patients, favorable outcomes were achieved by 72 (11.56%) at discharge and by 335 (53.78%) on the 90th day after stroke. A detailed distribution of patients into outcome groups based on mRS scores at both time points for both PMT and AMT is provided in Fig. 1.

We conducted an ordinal univariate logistic regression analysis to identify factors influencing patient functional status after MT, both at discharge and after 90 days. The following are the statistically significant factors affecting patients' conditions at discharge: age (OR: 0.957, 95% CI: 0.938–0.976, p < 0.001), female sex (OR: 0.614, 95% CI: 0.388–0.967, p = 0.036), NIHSS1 (OR: 0.853, 95% CI: 0.813–0.893, p < 0.001), DM (OR: 0.505, 95% CI: 0.286–0.865, p = 0.015), location of arterial occlusion: MCA (OR: 2.388, 95% CI: 1.040–5.521, p = 0.040) and PCA (OR: 4.873, 95% CI: 1.403–16.461, p = 0.011), mTICI 2b-3 (OR: 1.312, 95% CI: 0.660–1.813, p = 0.002), time from symptoms to MT (OR: 0.996, 95% CI: 0.993–0.998, p = 0.001), MT time (OR: 0.986, 95% CI: 0.980–0.992, p < 0.001), occurrence of ICH-PH2 (OR: 0.751, 95% CI: 0.147–1.513, p = 0.022) and performing a DH (OR: 0.217, 95% CI: 0.210–1.171, p = 0.037). Factors influenced outcomes on the 90th day after stroke were: age (OR: 0.960, 95% CI: 0.943–0.976, p < 0.001), NIHSS1 (OR: 0.894, 95% CI: 0.861–0.925, p < 0.001), AF (OR: 1.553, 95% CI: 1.029–2.359, p = 0.037), IVT (OR: 1.539, 95% CI: 1.051–2.262, p = 0.027), mTICI 2b-3 (OR: 0.792, 95% CI: 0.871–0.921, p = 0.002), MT time (OR: 0.989, 95% CI: 0.984–0.994, p < 0.001), performing a DH (OR: 1.214, 95% CI: 1.047–2.314, p = 0.026), and occurrence of ICH-PH2 (OR: 0.312, 95% CI: 0.311–0.864, p = 0.039). The entire analysis is presented in Table 2.

**Table 2:** Ordinal regression analysis of the influence of clinical phenodata on the favorable functional status (0–2 at mRS) of all patients at discharge and on 90th day of stroke.

Coefficients	At discharge			At 90th day after MT		
	OR	95% CI	p-value	OR	95% CI	p-value
Age	0.957	(0.938–0.976)	0.000	0.960	(0.943–0.976)	0.000
Female sex	0.614	(0.388–0.967)	0.036	0.919	(0.629–1.339)	0.661
NIHSS1	0.853	(0.813–0.893)	0.000	0.894	(0.861–0.925)	0.000
AF	1.374	(0.827–2.282)	0.219	1.553	(1.029–2.359)	0.037
Arterial hypertension	1.034	(0.616–1.754)	0.900	1.379	(0.893–2.140)	0.148
DM	0.505	(0.286–0.865)	0.015	0.759	(0.500–1.149)	0.193
Atherosclerosis	1.356	(0.839–2.192)	0.213	0.981	(0.660–1.461)	0.926
Smoking	1.034	(0.801–1.334)	0.798	0.984	(0.798–1.213)	0.879
Dyslipidemia	1.177	(0.770–1.792)	0.448	1.034	(0.733–1.458)	0.850
IVT	0.854	(0.541–1.351)	0.497	1.539	(1.051–2.262)	0.027
ICA	0.996	(0.230–4.259)	0.996	0.547	(0.161–1.826)	0.327
ACA	0.814	(0.200–2.712)	0.752	0.595	(0.210–1.669)	0.321
MCA	2.388	(1.040–5.521)	0.040	1.296	(0.649–2.616)	0.465
VA	0.643	(0.155–2.404)	0.524	0.497	(0.154–1.642)	0.243
BA	1.555	(0.486–4.759)	0.444	0.897	(0.338–2.396)	0.827
PA	4.873	(1.403–16.461)	0.011	1.556	(0.511–5.055)	0.444
mTICI 2b–3	1.312	(0.660–1.813)	0.002	0.792	(0.871–0.921)	0.002
Time from symptoms to MT	0.996	(0.993–0.998)	0.001	0.998	(0.996–1.000)	0.114
MT time	0.986	(0.980–0.992)	0.000	0.989	(0.984–0.994)	0.000
ICH	0.751	(0.147–1.513)	0.022	0.312	(0.311–0.864)	0.039
DH	0.217	(0.210–1.171)	0.037	1.214	(1.047–2.314)	0.026

*mRS* modified Rankin Scale, *OR* odds ratio, *CI* confidence interval, *NIHSS1* National Institutes of Health Stroke Scale on admission, *AF* atrial fibrillation, *DM* diabetes mellitus, *IVT* intravenous thrombolysis, *ICA* internal carotid artery, *ACA* anterior cerebral artery, *MCA *middle cerebral artery, *VA *vertebral artery, *BA* basilar artery, *PA *posterior cerebral artery, *mTICI 2b–3* modified Thrombolysis In Cerebral Infarction scale 2b–3, *MT* mechanical thrombectomy, *ICH* intracranial haemorrhage, *DH* decompresivehaemicraniectomy.

The multivariate analysis for all patients without subdivision into AMT and PMT subgroups, revealed that favorable outcomes, at discharge were influenced by DM (OR: 0.496, 95% CI: 0.301–0.795, p = 0.005), mTICI 2b-3 (OR: 1.145, 95% CI: 1.080–1.223, p < 0.001), occurrence of ICH-PH2 (OR: 0.503, 95% CI: 0.272–0.881, p = 0.021), time from symptoms to MT (OR: 0.997, 95% CI: 0.994–0.999, p = 0.004), and MT time (OR: 0.993, 95% CI: 0.987–0.998, p = 0.011). On the 90th day after stroke, outcomes were influenced by mTICI 2b–3 (OR: 1.095, 95% CI: 1.053–1.140, p < 0.001) and performing a DH (OR: 0.298, 95% CI: 0.109–0.689, p = 0.009). Table 3 displays the influence of clinical phenotypic data on functional status at discharge and 90 days after discharge.

**Table 3:** Ordinal regression analysis of the influence of clinical phenodata on favorable functional status (0–2 at mRS) at discharge and on the 90th day after the stroke.

Coefficents	OR	95% CI	P-value
At discharge			
DM	0.496	(0.301–0.795)	0.005
mTICI 2b–3	1.145	(1.080–1.223)	< 0.001
ICH	0.503	(0.272–0.881)	0.021
Time from symptoms to MT	0.997	(0.994–0.999)	0.004
MT time	0.993	(0.987–0.998)	0.011
at 90 day			
mTICI 2b–3	1.095	(1.053–1.140)	< 0.001
DH	0.298	(0.109–0.689)	0.009

Multivariate analysis. Only statistically significant results.

*mRS *modified Rankin Scale, *OR* odds ratio, *CI* confidence interval, *DM* diabetes mellitus, *mTICI 2b–3* modified Thrombolysis In Cerebral Infarction 2b–3, *ICH* intracranial haemorrhage, *MT* mechanical thrombectomy, *DH* decompressive heamicraniectomy.

The only statistically significant factor affecting the patient prognosis in the AMT subgroup, both on the day of discharge (OR: 1.796, CI 95%: 1.888–3.255, p = 0.017) and on the 90th day after the procedure (OR: 1.357, 95% CI: 1.025–1.851, p = 0.040), was mTICI 2b–3 score.

Statistically significant factors influencing the condition of patients after PMT on the day of discharge were: NIHSS2 (OR: 0.865, 95% CI: 0.840–0.890, p < 0.001), IVT (OR: 0.643, 95% CI: 0.479–0.855, p = 0.003), mTICI 2b-3 (OR: 1.058, 95% CI: 1.020–1.097, p = 0.003), MT time (OR: 0.993, 95% CI: 0.989–0.996, p < 0.001), and WBC count (OR: 0.961, 95% CI: 0.928–0.988, p = 0.014). On the 90th day after stroke there were: age (OR: 0.978, 95% CI: 0.966–0.990, p < 0.001), NIHSS2 (OR: 0.900, 95% CI: 0.879–0.921, p < 0.001), performing a DH (OR: 0.552, 95% CI: 0.315–1.008, p = 0.044), MT time (OR: 0.993, 95% CI: 0.990–0.997, p < 0.001), CRP concentration (OR: 0.995, 95% CI: 0.992–0.999, p = 0.015), and WBC count (OR: 0.974, 95% CI: 0.957–0.998, p = 0.013). The entire analysis is presented in Table 4.

**Table 4:** Ordinal regression analysis of the influence of clinical phenodata on favorable functional status (0–2 at mRS) patients with MT in vertebral-basil artery supply-at discharge and on the 90th day after stroke.

Coefficients	OR	95% CI	P-value
At discharge			
NIHSS2	0.865	(0.840–0.890)	0.000
IVT	0.643	(0.479–0.855)	0.003
mTICI 2b–3	1.058	(1.020–1.097)	0.003
MT time	0.993	(0.989–0.996)	0.000
WBC count	0.961	(0.928–0.988)	0.014
At 90thday after stroke			
Age	0.978	(0.966–0.990)	0.000
NIHSS2	0.900	(0.879–0.921)	0.000
DH	0.552	(0.315–1.008)	0.044
MT time	0.993	(0.990–0.997)	0.000
CRP concentration	0.995	(0.992–0.999)	0.015
WBC count	0.974	(0.957–0.998)	0.013

Multivariate analysis. Only statistically significant results.

*mRS *modified Rankin Scale, *OR* odds ratio, *CI* confidence interval, *NIHSS2 *National Institutes of Health Stroke Scale 24 h after procedure, *IVT* intravenous thrombolysis, *mTICI 2b–3* modified Thrombolysis In Cerebral Infarction 2b–3, *MT* mechanical thrombectomy, *WBC *white blood cell, *DH* decompressive haemicraniectomy.

## Discussion

### The differences between AMT and PMT groups of patients

In our single-center study, we looked for any differences between the groups of patients who underwent AMT and PMT.

Age emerged as a significant parameter: the median age of AMT patients was higher than that of PMT patients (average age: 70 vs. 63 years, p < 0.001). Similar disparities have been observed in previous studies^10,14^. In addition, women constituted a smaller proportion of the study population (44.94%), with a higher percentage in the AMT group (AMT: 46.5%, PMT: 24.6%, p = 0.001). This observation also aligns with findings from other studies^10,14^. Furthermore, both age and gender distribution were also relevant in a meta-analysis that analyzed data from 16 studies^9^.

The NIHSS score both before and 24 h after procedure were significantly higher in the AMT group than in the PMT group (NIHSS1; mean: 13 vs. 9, p < 0.001 and NIHSS2; mean: 12 vs. 7, p = 0.006). These results imply that patients who underwent AMT initially presented with more severe neurological symptoms and were in a poorer overall condition compared to patients with PMT. Similarly, in a study by Schlemm et al.^14^, patients with PMT had lower NIHSS2, whereas other results are presented in the literature in which NIHSS scores were significantly higher in the PMT group^7–9^.

The proportion of patients with a favorable prognosis was comparable in both study groups. These results are largely consistent with other reports^8–11,15^. However, Schlemann et al. obtained different results: PMT patients exhibited significantly worse mRS scores both at discharge and on the 90th day^14^. In a meta-analysis by Xun et al., in a subgroup analysis that included only studies with large groups, PMT was also associated with a worse mRS score on the 90th day after procedure^16^.

In our study, we did not find any differences in mortality between the two groups, this was also found to be the case in a study by Jahan et. al^10^. Nevertheless, increased mortality was observed in patients after PMT in both individual studies^7,8,15^and meta-analyzes^9,16^.

We found no significant distinctions in reperfusion efficiency (mTICI 2b–3), however the number of successful reperfusions was lower in the PMT group, this has also been found by other authors^7,8,10,14^. Additionally we did not identify any significant differences in the duration of the MT; instead, the average duration of the procedure in the PMT group was slightly longer, as reported by other authors^8^.

Strokes in the posterior vasculature are often linked to increased diagnostic challenges^17^ and a higher likelihood of misdiagnosis^18^. Therefore, the time from symptom onset to procedure might be longer in PMT compared with AMT. However, we did not observe such an association, in our groups, this is consistent with findings from other studies^7,10,14^. A meta-analysis demonstrated a significant difference in the time interval from symptom onset to the procedure, favoring AMT^9^.

### Factors related with outcomes after MT

In our patients, those of a younger age showed significantly better functional outcomes (OR: 0.957, 95% CI: 0.938–0.976 at discharge and OR: 0.960, 95% CI: 0.943–0.976 on the 90th day). Older age is a well-established factor associated with a poorer prognosis following MT. Numerous studies have consistently demonstrated that older patients have a reduced likelihood of achieving a good functional status and experience higher mortality rates compared to their younger counterparts^19–24^. This effect was also pronounced purely in patients after PMT, both in our results (OR: 0.978, 95% CI: 0.966–0.990, for favorable outcomes on the 90th day) and in those of other authors^25–27^. On the other hand, influence of age on the outcomes of MT is not necessarily clear; Sweid et al. performed a study comparing outcomes in the individuals over and under 90 years of age and found no significant differences between the groups, even reporting a lower mortality rate in the elderly group (11.54% vs. 13.06%, p = 0.82)^28^.

The most important predictor of a bad prognosis in our study was the NIHSS score on admission; a higher score was associated with poorer outcomes both at discharge and on the 90th day after intervention (OR: 0.853, 95% CI: 0.813–0.893 and OR: 0.894, 95% CI: 0.861–0.925 respectively). These results are consistent with reports from previous studies^10,15,20–24,29,30^ and imply the conclusion, that the milder the stroke is, the better outcomes could be achieved. Moreover, both the literature reports and our results concur that a higher NIHSS score is associated with a less favorable prognosis particularly in PMT, for both short-term (OR: 0.865, 95% CI: 0.840–0.890) and long-term (OR: 0.900, 95% CI: 0.879–0.921) outcomes^25–27,31^. Furthermore, Kniep et al.^26^ showed that the 24-h NIHSS score ≤ 9 best serves as a surrogate for a favorable outcome (0–2 mRS) on the 90th day after PMT (AUC: 0.89, 95% Cl: 0.85–0.92).

The occurrence of hemorrhagic transformation after MT is associated with early neurologic impairment and a lack of subsequent neurological improvement^30^, this decreases the chances of achieving a good prognosis^22,29,30^, and is linked to higher mortality^22^. In our study, intracranial hemorrhage was identified as a factor associated with a poorer prognosis, with the strongest influence observed at discharge (univariate OR: 0.751, 95% CI: 0.147–1.513, multivariate OR: 0.503, 95% CI: 0.272–0.881), this is consistent with previous reports . In a study performed by Huang et al.^21^ it was demonstrated that postoperative hemorrhage transformation was partly responsible for the worse mRS score on the 90th day in patients with prolonged MT time. Furthermore, in recent studies reporting only on patients with posterior circulation strokes, the incidence of ICH was significantly higher in the group of patients undergoing PMT than in the control group^2,3^.

Successful recanalization, served as a positive predictor of a favorable prognosis at discharge following PMT (OR: 1.058, 95% CI: 1.020–1.097), and exhibited even greater predictive power following AMT (OR: 1.769, 95% CI: 1.888–3.255 at discharge and OR: 1.357, 95% CI: 1.025–1.851 on the 90th day). The same results have been obtained in many other studies^21,22,24,29,32^. Successful recanalization was a pivotal component of an effective therapeutic process in PMT, as corroborated by previous research^31^, and its absence led to increased mortality on the 90th day after the procedure^25^. Similarly, across both our groups, a higher mTICI score was associated with better outcomes at discharge (univariate OR: 1.312, 95% CI: 0.660–1.813 and multivariate OR: 1.145, 95% CI: 1.080–1.223) and on the 90th day (multivariate OR: 1.095, 95% CI: 1.053–1.140). Factors such as older age, higher baseline NIHSS score, pre-stroke mRS score, and general anesthesia are thought to be the risk factors for unsuccessful recanalization^32–34^.

The duration of the procedure > 60 min is another recognized risk factor for a worse prognosis^11,22,29^, as is prolonged time from symptom onset to procedure^30^. In our study, these associations were confirmed, especially at the discharge time point; a shorter time from symptoms to MT (univariate OR: 0.996, 95% CI: 0.993–0.998, multivariate OR: 0.997, 95% CI: 0.994–0.999) and a shorter procedure duration (univariate OR: 0.986, 95% CI: 0.980–0.992, multivariate OR: 0.993, 95% CI: 0.987–0.998) all indicated a better patient outcome. In addition, longer procedure duration was found to be significant, particularly in the PMT subgroup, and worse patient outcomes than in the overall analysis, both at discharge (OR: 0.993, 95% CI: 0.989–0.996) and at day 90 after the procedure (OR: 0.993, 95% CI: 0.990–0.997). Therefore, to the best of our knowledge, the influence of this factor, especially in the PMT group, has not yet been documented in the literature.

The presence of AF, as the comorbidity (OR: 1.533, 95% CI: 1.029–2.359) positively affected patient prognosis on the 90th day. The positive effect of AF on patient outcome after MT has been previously noted^30,35^, however, there are also studies indicating a negative effect of AF^22,36^, as well as the absence of an effect^16,24,37^. These discrepancies may be explained by the frequent use of anticoagulants in this disease, which may facilitate recanalization and, on the other hand, increase the likelihood of a bleeding complication.

The presence of DM had a detrimental effect on discharge prognosis (univariate OR: 0.505, 95% CI: 0.286–0.865, multivariate OR: 0.496, 95% CI: 0.301–0.795), which is also confirmed by the literature^16,22,29,30^. Diabetic microangiopathy and the hyperglycemic environment contribute to poor development of collaterals^38^. Due to insulin resistance, diabetes can also lead to endothelial damage, coagulation-fibrinolysis balance and platelet activity^39^.

### Factors influencing outcomes particularly in PMT group.

In a recent study focusing on patients with posterior circulation strokes, Tao et al.^2^ demonstrated that PMT is associated with better patients' functional status (for 0–2 at mRS, adjusted risk ratio: 3.17, 95% CI: 1.84–5.46) and lower mortality (adjusted risk ratio: 0.66; CI 95%: 0.52–0.82) compared to the best conservative treatment (with or without IVT). In addition, another study showed that PMT is more beneficial even in an extended time window of 6–24 h^3^. Considering these results, the discussion about factors influencing outcomes exclusively in the PMT group is even more essential.

Leukocytosis and a high CRP concentration, both indirect indicators of inflammatory processes in the body, negatively impacted patient outcomes at both discharge and on the 90th day following PMT (OR: 0.961, 95% CI: 0.928–0.988, and OR: 0.974, 95% CI: 0.957–0.998 for WBC, and OR: 0.995, 95% CI: 0.992–0.999 for CRP concentration). These results suggest a robust correlation between the severity of post-MT inflammation and the likelihood of achieving a favorable prognosis, particularly in patients with PMT. This association was not observed in the overall MT patient group. The adverse effects of leukocytosis, elevated CRP levels, and other inflammatory markers, such as IL-6, on a prognosis following stroke have been well-documented in the general stroke population^40,41^, and in patients after MT in particular^23,42,43^. A similar association was observed for leukocytosis, which also increased both mortality (multivariate OR: 1.86, CI 95%: 1.07–2.63) and the odds of a poor outcome (mRS < 2, multivariate OR: 1.51, CI 95%: 0.51–4.60), alike without differentiation by AMT and PMT. Additionally, contemporary research has placed increasing importance on the systemic immune inflammation index, which has proven to be an effective tool for predicting poor outcomes after stroke^44,45^.

IVT is the other factor which has an effect on post-stroke functional state. In our group, IVT showed a positive effect on the prognosis of patients (OR: 1.539, 95% CI: 1.051–2.262). Despite the fact that studies suggest a better prognosis and lower mortality when combined with IVT compared to MT alone^46,47^, they often do not specify this effect exclusively for PMT. Additionally, the literature consistently reports a lower risk of bleeding complications in PMT compared to AMT^48,49^. However, the Capellari study revealed that the combination of IVT and MT was associated with a significantly higher rate of ICH (ECASS II) compared to IVT alone (3.1% vs 1.9%; OR 3.984, 95% CI 95%: 1.014–15.815), and the likelihood of a favorable outcome varied depending on the affected artery^30^. Another study focusing exclusively on patients with posterior artery occlusion, demonstrated an increased risk of bleeding and higher mortality after endovascular treatment, with or without IVT, compared to IVT alone^50^, but also indicated an increased chance of an excellent outcome in the MT group (OR: 1.50, 95% CI: 1.07–2.09). Moreover, Lee et al.^48^ in the meta-analysis showed worse outcomes following IVT in the PMT group compared to the AMT group.

The performance of a DH was a factor that significantly reduced the odds of a favorable prognosis by nearly half in the PMT group (OR: 0.552, 95% CI: 0.315–1.008) and by approximately 70% in our overall study population (OR: 0.298, 95% CI: 0.109–0.689). While the impact of DH on outcomes following malignant middle cerebral artery infarction (mMCAi) remains a subject of debate, there is a dearth of literature that reports on its effects after posterior vascular strokes. In one study of DH after IVT, no patient over 60 years old survived to 3-month follow-up, and moderate disability at 3 months was present in 35.5%^51^. Inamasu et al. showed that despite the absence of differences in any of the demographic variables evaluated, the mortality rate was significantly higher in the group of individuals over 70 years old who underwent DH compared to those aged 61–70^52^.

Our results confirmed the co-existence of the some parameters (sociodemographic, clinical and technical) influencing the final effect of MT. We assumed the differences depending on the location of the intervention and finally identified two key parameters for the effect in posterior circulation interventions. Our results require confirmation in randomised clinical trials and may optimise MT management in the future.

Understanding the relevance of these parameters in MT outcomes is crucial in guiding the selection of patients based on individual factors to optimize the clinical efficacy of MT, both in cases involving the anterior and posterior cerebral circulation.

### Limitations of the study

We have several limitations to acknowledge. Firstly, patients who underwent MT were either directly admitted to our center or transferred from a primary center following the drip-and-ship model. This situation could introduce variations in qualification for MT, and the time elapsed between MT and IVT administration. Secondly, the admission and qualification for MT were conducted by different physicians, potentially resulting in variations in the interpretation of clinical and radiologic symptoms. Thirdly, NIHSS Scale was utilized to evaluate the clinical condition of patients undergoing AMT and PMT procedures. It is worth noting that the NIHSS scale may not comprehensively capture the severity of the clinical condition in patients with posterior circulation stroke^51^. Fourthly, our study primarily relied on the clinical status of patients at discharge and on the 90th day after the procedure, whereas some investigations extend their focus to outcomes after one year. Finally, the retrospective nature of our study and the relatively small size of the subgroup of patients undergoing PMT may potentially compromise the robustness of our results. Moving forward, it is imperative to undertake prospective studies encompassing a larger number of patients with posterior cerebrovascular stroke to further advance our understanding in this area.

## Conclusions

Our study revealed distinct clinical profiles between patients who underwent AMT and PMT. Several sociodemographic and clinical factors were found to influence the recovery of stroke patients undergoing MT. Notably, the neurological status one day after MT emerged as the most crucial prognostic parameter for achieving a favorable functional status in both groups. Furthermore, within the PMT group, factors such as the timing of the procedure and the concentrations of inflammatory markers appeared to play pivotal roles in predicting a favorable outcome. These factors could serve as both predictive indicators and targets for modification to enhance the likelihood of a better prognosis in this specific patient group.

## Data Availability

The datasets generated and analyzed during the study are not publicly available because they contain personal data of the patients, but they can be requested from the corresponding author upon reasoned request.
